# Monitoring of cases of anosmia and COVID-19

**DOI:** 10.1016/j.bjorl.2021.04.007

**Published:** 2021-05-05

**Authors:** Pathum Sookaromdee, Viroj Wiwanitkit

**Affiliations:** a Private Academic Consultant, Bangkok, Thailand; b Dr DY Patil University, Pune, India

Dear Editor,

We would like to share ideas on the publication “Monitoring of cases of anosmia may help control the COVID-19 pandemic.^1^” Miyake et al. concluded that “The adoption of a unified internet self-reported notification system of new cases of sudden dysosmia ….. this pandemic”.^1^ In fact, asymptomatic and atypical clinical presentation is not uncommon in COVID-19 and it is necessary that the practitioner has to recognize for the possibility.^2^, ^3^ Anosmia is a possible clinical presentation in COVID-19. Screening for anosmia might be difficult but it is useful for early detection of the case.^4^ A reporting tool might be helpful for screening.^5^ Regarding the self-reported notification system, an important consideration is on the reliability. In a developing country, a low educated people might disguise the clinical history and the reporting system is usually not useful.^6^ A non-subjective screening tool should be considered for anosmia screening.

## Conflicts of interest

The authors declare no conflicts of interest.
